# Antimalarial and immunomodulatory potential of chalcone derivatives in experimental model of malaria

**DOI:** 10.1186/s12906-022-03777-w

**Published:** 2022-12-12

**Authors:** Shweta Sinha, Bikash Medhi, B. D. Radotra, Daniela I. Batovska, Nadezhda Markova, Ashish Bhalla, Rakesh Sehgal

**Affiliations:** 1 Department of Medical Parasitology, Post Graduate Institute of Medical Education and Research, Chandigarh, 160012 India; 2 Department of Pharmacology, Post Graduate Institute of Medical Education and Research, Chandigarh, India; 3 Department of Histopathology, Post Graduate Institute of Medical Education and Research, Chandigarh, India; 4 Institute of Organic Chemistry With Centre of Phytochemistry, Bulgarian Academy of Sciences, Sofia, Bulgaria; 5 Department of Internal Medicine, Post Graduate Institute of Medical Education and Research, Chandigarh, India

**Keywords:** Malaria, In vivo, *P. berghei*, ICAM-1, Chalcones, Cytokine expression

## Abstract

**Background:**

Malaria is a complex issue due to the availability of few therapies and chemical families against Plasmodium and mosquitoes. There is increasing resistance to various drugs and insecticides in Plasmodium and in the vector. Additionally, human behaviors are responsible for promoting resistance as well as increasing the risk of exposure to infections. Chalcones and their derivatives have been widely explored for their antimalarial effects. In this context, new derivatives of chalcones have been evaluated for their antimalarial efficacy.

**Methods:**

BALB/c mice were infected with *P. berghei* NK-65. The efficacy of the three most potent chalcone derivations (1, 2, and 3) identified after an in vitro compound screening test was tested. The selected doses of 10 mg/kg, 20 mg/kg, and 10 mg/kg were studied by evaluating parasitemia, changes in temperature, body weights, organ weights, histopathological features, nitric oxide, cytokines, and ICAM-1 expression. Also, localization of parasites inside the two vital tissues involved during malaria infections was done through a transmission electron microscope.

**Results:**

All three chalcone derivative treated groups showed significant (*p* < 0.001) reductions in parasitemia levels on the fifth and eighth days of post-infection compared to the infected control. These derivatives were found to modulate the immune response in a *P. berghei* infected malaria mouse model with a significant reduction in IL-12 levels.

**Conclusions:**

The present study indicates the potential inhibitory and immunomodulatory actions of chalcones against the rodent malarial parasite *P. berghei*.

**Supplementary Information:**

The online version contains supplementary material available at 10.1186/s12906-022-03777-w.

## Background

Malaria is a worldwide infectious illness that continues to be a major cause of morbidity and mortality in developing countries. *Plasmodium falciparum* is the most common *Plasmodium* species that causes deadly malaria [1]. *P. falciparum* infection leads to a significant number of blood-stage parasites and is responsible for modifying the surface of the infected red blood cell (RBC) by creating an adhesive phenotype, e.g., “sticky cell,” causing RBC sequestration inside small and medium-sized vessels. Sequestration leads to splenic parasite clearance avoidance, host cell endothelial damage, and microvascular blockage [2, 3]. The host's immune system releases a number of proinflammatory molecules during the blood infection stage in response to the parasite's presence, including IL-1, IL-6, IL-8, IL-12 (p70), IFN-γ, and TNF, all of which play a key role in limiting the parasite's growth and removal [4]. According to WHO report 2021, malaria cases has been increased from 227 to 241 million in the year 2020 [5]. The global attempt to eradicate malaria began in the 1950s, but it failed due to mosquito resistance to the insecticides employed, malaria parasite resistance to the drug used in treatment, and administrative challenges [6]. Efforts to develop an efficient antimalarial vaccine, as well as clinical trials, are underway. Furthermore, in Southeast Asia, *P. falciparum* resistance to artemisinin derivatives, piperaquine, and mefloquine indicates that novel antimalarials are urgently needed. The process of identifying new antimalarials, dose-finding, and evaluation has also evolved over the last 15 years [7]. Most of the agents that are presently under clinical development are blood schizonticides for the treatment of uncomplicated *falciparum* malaria, under evaluation either singly or as part of two-drug combinations [8], as they act on the asexual forms in the erythrocytes and interrupt clinical attacks and are also easier to manipulate in the laboratory. Nonetheless, malaria mouse models are a simple way to evaluate the in vivo effects of potentially beneficial antimalarial drugs and are commonly used in antimalarial compound screening [9].

Furthermore, natural products with a wide range of chemical structures, such as alkaloids, chalcones, steroids, terpenes, quinones, flavonoids, coumarines, naphthopyrones, xanthones, polyketides, phenols, peptides, lignans, chomenes, and others, have been extensively investigated as antimalarial drugs [10]. Chalcones (1,3-diaryl-2-propen-1-ones) are precursors to flavonoids and isoflavonoids, which can be found in many edible plants. Chalcone derivatives have been reported to have distinct pharmacological activities, such as anticancerous, antimicrobial, anti-HIV, antimalarial, and antinociceptive activities [11–15]. Chalcones have a vast number of bioactive molecules with a wide range of molecular targets. Even slight structural alterations in chalcones can cause them to target different biological functions. Furthermore, these chalcones have been shown to inhibit tumour cell invasion and metastasis in vitro by targeting one or more molecules such as NF-kB, TNF, VEGF, ICAM-1, VCAM-1, bcl-2, and MMP [16–18], and it has been reported that chalcone derivatives inhibit secretory phospholipase A2, COX, lipoxygenases, proinflammatory cytokines production, neutrophil chemotaxis, phagocytosis, and production of reactive oxygen species (ROS) [19, 20]. Additionally, a number of in vitro studies [21–26], previously been carried out on both chloroquine sensitive and chloroquine resistant strains that show chalcones have immense antimalarial potential. However, there were only a few studies that showed antimalarial activity of chalcones in both in vitro and in vivo malaria models. Chen et al. [27], described 2,4-Dimethoxy-4'-Butoxychalcone as a new antimalarial drug with excellent antimalarial activity in both in vitro and in vivo malaria models with no toxicity. It inhibited [3H]hypoxanthine absorption in chloroquine-susceptible (IC_50_ of 3D7 was 8.9 mM) and chloroquine-resistant (IC_50_ of Dd2 was 14.8 mM) *P. falciparum* strains in a concentration-dependent manner. This compound extremely suppressed the parasitemia when given orally and intraperitoneally at a dose of 50 mg/kg/day and subcutaneously at a dose of 20 mg/kg/day for 5 continuous days, and protected *P. berghei* K173 infected mice from deadly illness. In an another study, 1, 1-*Bis*-[(3′,4′-*N*-(urenylphenyl)-3-(3″,4″,5″-trimethoxyphenyl)]-2-propen-1-one, identified as the most active by in vitro tests, and was tested in mice infected with *P. berghei* (ANKA), a chloroquine-susceptible strain of murine malaria. This compound was able to decrease the parasitemia and delay the progression of malaria but did not eradicate the infection [28]. So, with these rationales, the present study primarily aimed to find antimalarial potential in a malaria mouse model and was further extended to find whether these chalcones can modulate the immune response or not.

## Methods

### Experimental animals

The present study was carried out at the Postgraduate Institute of Medical Education and Research (PGIMER), Chandigarh and reported in accordance with the ARRIVE guidelines. The study was conducted after approval from the Institutional Animal Ethics Committee Ref. No. 69/IAEC/418 as per the Committee for the Purpose of Control and Supervision of Experiments on Animals (CPCSEA) guidelines and the Institute Bio-Safety Committee Ref. No. 04/IBC/2013. The inbred BALB/c mice, 6–8 weeks old, weighing between 20–32 g of either sex and 4–6 week old Swiss mice, weighing between 20–28 g, were procured from the Advanced Facility for Small Animal Research, PGIMER, Chandigarh. Until the end of the experiments, the animals were kept in polypropylene cages with conventional laboratory settings, including a constant temperature of 25 °C and 12 h light/dark cycles. Animals were given free access to a mouse chow diet and water in a room. To achieve meaningful statistical results, we used a minimally sufficient number of animals in all cases. All procedures conducted on the animals were in accordance with the rules and regulations as set out by the CPCSEA guidelines. After experimental procedures were over, each animal was sacrificed by giving anaesthesia followed by cervical dislocation. This euthanasia procedure was done according to CPCSEA guidelines.

### Drugs and chemicals

The chalcones were synthesised at the Institute of Organic Chemistry with Centre of Phytochemistry, Bulgarian Academy of Sciences, Sofa, Bulgaria, as described in previous study [29]. The three chalcone derivatives namely, (E)-1-(2,5-Dimethoxyphenyl)-3-(4-methoxyphenyl)prop-2-en-1-one, (1); (E)-(3,4,5-Trimethoxyphenyl)-3-(4-methoxyphenyl)prop-2-en-1-one, (2); and. (E)-1-(3,4,5-Trimethoxyphenyl)-3-(3,4-dimethoxyphenyl)prop-2-en-1-one,(3), were screened for potent antimalarial effect shown formerly by our group [29, 30].

Apart from these derivatives, chloroquine phosphate and Griess reagent were purchased from Sigma-Aldrich, USA. ICAM-1 [Anti-ICAM1 antibody [YN1/1.7.4] from ABCAM, USA and the BD CBA Mouse Soluble Protein Flex Set System for IL-1, IL-6, TNF-alpha, IFN-γ, IL-10 and IL-12p70 were purchased from BD Biosciences, USA. All other chemicals and reagents used in this study were of analytical grade.

### Parasites and experimental models validation

*Plasmodium berghei* NK-65 was procured from Punjab University, Chandigarh and was maintained in Swiss albino mice by serial passage by intraperitoneal injections of 0.2 mL of infected blood suspension containing 1 × 10^6^ parasitized red blood cells (pRBC) every 10 days. Furthermore, this strain was used in model validation.

Inbred BALB/c, 6–8-week-old mice were inoculated by intraperitoneal injection of 0.2 mL of infected blood suspension containing 1 X 10^7^ *P. berghei* NK-65 (chloroquine sensitive strain) infected erythrocytes. Each mouse was checked for any parasitemia, physical signs, and mortality. The parasitemia in all the infected mice was evaluated using a thin smear of peripheral blood taken from the tail followed by Giemsa staining (10% solution in phosphate buffer, pH 7.2) and visualised under a light microscope with 1000X magnification [31]. The final confirmation of model validation was done through histopathological study on six major organs, i.e., liver, spleen, heart, lungs, brain, and kidneys, which were collected on day 8 post-infection from each mouse (Figure S1).

### Dose selection studies

Doses of each chalcone derivative 1 (10 mg/kg), 2 (20 mg/kg), and 3 (10 mg/kg) were obtained after extrapolation of in vitro data published in our previous study [29], and the route of administration of these derivatives, i.e., derivative 1 was given through the intraperitoneal route, and derivatives 2 and 3 through the oral route, were chosen from the pharmacokinetic study [32].

### Experimental design

**Fig. 1:**
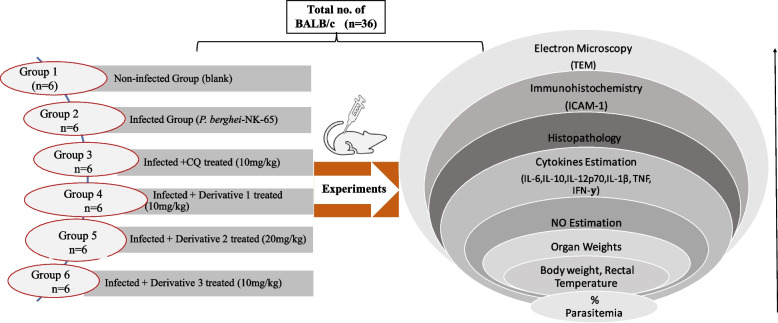
Experimental design of the study

A total of thirty-six BALB/c mice were recruited for the main experiment. These mice were randomly divided into six groups (Groups 1–6). Each group consists of six mice (Fig. 1).

Group 1: (Non-infected): Injected intraperitoneally with blank media (PBS).

Group 2: (Infected): Inoculated by intraperitoneal injection with 1 × 10^7^ *P. berghei* NK-65 infected erythrocytes.

Group 3: Infected mice treated with CQ.

Group 4: Infected mice treated with Chalcone derivative 1.

Group 5: Infected mice treated with Chalcone derivative 2 and,

Group 6: Infected mice treated with Chalcone derivative 3.

### Treatment procedure

For the intraperitoneal/oral administration, a suspension formulation of screened chalcones was prepared by triturating the weighed quantity in a dry mortar with 0.5% carboxymethyl cellulose (CMC) and was prepared by drop-wise addition of water and proper grinding, and chloroquine (CQ) was dissolved in PBS (pH 7.2). For all drug/derivatives treated groups, the first administration of the drug/derivatives was started on the 3^rd^ day of post-infection, which was continued till the 7^th^ day following standard Rane’s test procedure [33]. Thin blood smears were then made from the tail blood of each mouse every day, and parasitemia in mice was assessed from Giemsa stained smears.

### Blood parasitemia

Parasitemia was monitored by light microscope examination under (1000X) magnification using Giemsa-stained blood smears on the 3^rd^, 5^th^, and 8^th^ days after inoculation. The percentages of parasitemia were calculated by counting the number of parasite-infected erythrocytes per 2000 erythrocytes according to the equations below [34].

$$\mathbf{Percent}\boldsymbol\;\mathbf{Parasitemia}\boldsymbol\;\boldsymbol=\boldsymbol\;\boldsymbol(\mathbf{No}\boldsymbol.\boldsymbol\;\mathbf{infected}\boldsymbol\;\mathbf{RBCs}\boldsymbol\;\boldsymbol\div\boldsymbol\;\mathbf{Total}\boldsymbol\;\mathbf{No}\boldsymbol.\boldsymbol\;\mathbf{RBCs}\boldsymbol\;\mathbf{counted}\boldsymbol)\boldsymbol\;\boldsymbol\times\boldsymbol\;\mathbf{100}$$

### Temperature and body weight

The rectal temperature and body weight of each mouse in all the groups were measured before infection (day 0) and on the 3^rd^, 5^th^, 7^th^, and 8^th^ days of post-infection [34].

### Organ weight

The wet weight of two organs, i.e., the liver and spleen of each mouse in all the groups, was measured after sacrifices post-treatment.

### Serum nitric oxide estimation

At the end of the study, the blood samples from each mouse were withdrawn by tail vein. Serum was separated and the nitric oxide levels were measured in serum samples of mice. Nitrite is estimated using the Greiss reagent and serves as an indicator of nitric oxide production. Briefly, 100 µL of Greiss reagent (1:1 solution of 1% sulphanilamide in 5% phosphoric acid and 0.1% napthylamine diamine dihydrochloric acid in water) was added to 100 µL of serum, and the absorbance at 546 nm was measured after 15 min [35]. The nitrite concentration was calculated using a standard curve for sodium nitrite. Nitrite levels were expressed as µg/mL.

### Cytokines estimation

Whole blood was collected in sterile tubes at the post-treatment stage and allowed to coagulate for 2–3 h at 40◦C and then subjected to centrifugation. The sera thus obtained were stored at -80ºC until cytokine measurement was performed by using the BD CBA Mouse Soluble Protein Flex Set System for IL-1, IL-6, TNF-, IFN-γ, IL-10, and IL-12p70 following the manufacturer’s recommendations.

### Histopathology

Liver and spleen from all experimental groups were removed aseptically after being fixed *in toto* for 48 h in a neutral buffered 10% formalin solution. The organs were cut into serial cross sections and fixed in formalin for another 12 h. Finally, tissue sections were fixed in paraffin and cut into 5 mm thick sections, which were stained with haematoxylin and eosin and looked at for inflammation and parasites [36].

### Immunohistochemistry

Tissue sections of the liver and spleen were immunostained as described previously [37]. The Avidin–Biotin–peroxidase complex (ABC) technique was followed using primary antibodies against ICAM-1 antigen (product name: Anti-ICAM1 antibody [YN1/1.7.4]): Rat monoclonal antibody to ICAM-1 with species reactivity to mouse. Briefly, 3 µm sections were incubated overnight at 37 °C for 2 h with 1: 200 diluted rat monoclonal antibodies directed against murine ICAM-1. After washing, sections were incubated for 1 h at room temperature with biotin-conjugated secondary antibody (UltraTek HRP (Anti-Polyvalent), USA) ready to use. Peroxidase activity was developed in 0.5% 3, 3’-diaminobenzidine hydrochloride till the desired stain intensity was developed. Sections were washed in de-ionised H_2_O for 5 min, and slides were dried and mounted with DPX.

### Transmission electron microscopy

Ultrastructure changes after the treatment can be visualised by electron microscopy as described previously [38]. The specimens of liver and spleen of approximately 0.5 mm^3^ were fixed in 3% glutaraldehyde-0.1 M sodium phosphate buffer (pH 7.2), for 2 h at 4 °C. All samples were rinsed three times and kept overnight at 4 °C in a 0.2 M sucrose-0.1 M phosphate buffer (pH 7.2). The samples were then post-fixed in 2% OsO_4_ in 0.1 M phosphate buffer, washed twice in 0.1 M phosphate buffer and twice in distilled water, dehydrated in a graded alcohol series and then in propylene oxide, and embedded in Epon. Using a Reichert-OmU-2 ultramicrotome and a glass or diamond knife, thin slices were cut and deposited on Formvar carbon-coated grids. The sections were poststained with 6% aqueous uranyl magnesium acetate and Reynold’s lead citrate. The section was then examined with a Phillips 200 or 400 transmission electron microscope at 60 or 80 kV, according to the thickness of the section and the required magnification.

### Statistical analysis

Data is shown as mean ± SEM and analysed using the one-way analysis of variance (ANOVA), followed by the Bonferroni multiple comparison test. The analysis was performed using SPSS Version 16.0 software and *p* < 0.05 was taken as the level of significance. For evaluating the ICAM-1 expression, the Chi-square, contingency co-efficient, and correlation tests were applied.

## Results

Using the method described by Ryley and Peters (1970) [39], the schizonticidal activity of screened chalcone derivatives on established infection was evaluated. Thirty BALB/c mice were infected with *P. berghei* CQ-sensitive NK-65 parasitized RBCs by intraperitoneal injection on the first day (D0). Seventy-two hours later (D3), the mice were randomly divided into five groups of six mice each, and one more additional group of six mice without infection. Three groups of mice were treated with chalcone derivative 1 (10 mg/kg, intraperitoneally), derivative 2 (20 mg/kg, orally), and derivative 3 (10 mg/kg, orally), respectively. The negative control group was treated with PBS, while the positive control group was treated with CQ (10 mg/kg), respectively. Each chalcone derivative and the standard drugs were treated once daily for five days, i.e., from day 3 to day 7. Giemsa stained thin smears were prepared from tail blood samples collected on day 3^rd^, 5^th^, and on 8^th^ days post-infection to monitor parasitemia level. The body weight and rectal temperature were recorded on the 3^rd^, 5^th^, 7^th^ and 8^th^ days of post-infection.

### Blood parasitemia level, rectal temperature, body weights and organs weight

**Fig. 2:**
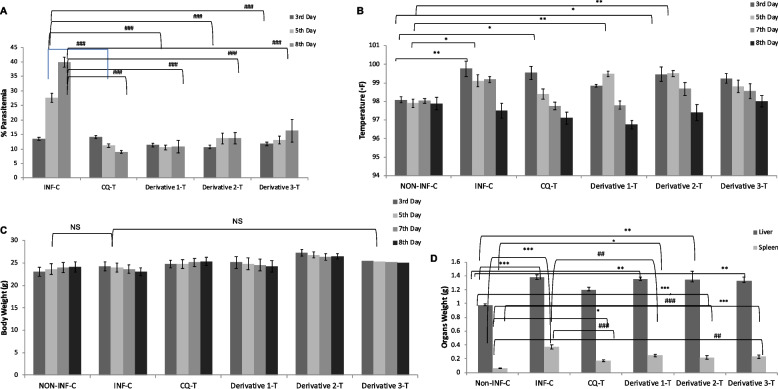
Effect of Chalcone derivatives 1 (10 mg/kg), 2 (20 mg/kg) and 3 (10 mg/kg) using Chloroquine diphosphate (10 mg/kg) as a standard chloroquine sensitive antimalarial drug for evaluating parasitemia (%) (parasitized RBC count) (**A**), on rectal temperature (◦F) (**B**), on body weights (g) (**C**) and, organ weights (g) (**D**) in *P. berghei* NK-65 infected mouse model. Data are represented as mean ± SEM, *n* = *6* mice. *p < 0.05, **p < 0.01, ***p < 0.001 (*Group represents INF-C, CQ-T, Chalcone derivatives 1, 2 and 3 compared to NON-INF-C Control group) and ^#^*p* < 0.05, ^# #^ *p* < 0.01, ^# # #^ *p* < 0.001 (^#^Group represents CQ-T, chalcone derivatives 1, 2, and 3 compared to infected Control group); NS: non-significant. Abbreviation Used: NON-INF-C: Non-infected control, INF-C: Infected Control, CQ-T: Chloroquine Treated

On established infection, it was observed that there was a daily increase in the parasitemia level of the infected-control group and vice-versa, i.e., a daily reduction in the percentage parasitemia of the CQ-treated group. All three chalcone derivative treated groups showed a significant (*p* < 0.001) reduction in parasitemia levels on the 5^th^ and 8^th^ days of post-infection, including the CQ-treated group compared to the infected control. The percentage parasitemia of derivative 1, 2, 3, and CQ-treated groups were 10.57 ± 2.12, 13.68 ± 1.98, 16.31 ± 3.88, and 9.04 ± 0.55 on the 8^th^ day post-infection as compared to the infected control (39.9 ± 1.8), (Fig. 2A).

The rectal temperature of the infected control (*p* < 0.01), CQ, and derivative 2 treated groups significantly (*p* < 0.05) increased on the 3^rd^ day of infection, and a significant (*p* < 0.01) increase was also shown in derivative 1 and derivative 2 in temperature on the 7^th^ day post-infection compared to the non-infected control (Fig. 2B).

Non-infected control and CQ-treated progressively gained weight from day 3^rd^ (23.06 ± 1.05) and (24.8 ± 1.16) till day 8^th^ (24.1 ± 0.87) and (25.36 ± 0.93), whereas a gradual decrease in body weight was observed in infected mice, derivative 1, 2, and 3 treated groups (Fig. 2C). Also, when comparing the treatment group to the non-infected control group and the infected control group, there was no significant weight gain or weight loss.

The wet weights of two organs, the liver and spleen, recorded a gradual increase towards the late stages of the infection as compared to the controls. The weight of the liver increased significantly (*p* < 0.001 and *p* < 0.01) in the infected control (1.38 ± 0.041) and in the derivatives 1 (1.35 ± 0.03), 2 (1.34 ± 0.12), and 3 (1.33 ± 0.05) treated groups as compared to the non-infected control (0.97 ± 0.02) except for the CQ (1.2 ± 0.3) treated group. Similarly, as compared to the non-infected control group, there was a significant (*p* < 0.001) increase in the weight of the spleen of the infected control, derivative 1, 2, and 3 treated groups. However, a significant (*p* < 0.01) reduction in the weight of the spleen was observed in all treated groups as compared to the infected control (Fig. 2D).

### Nitric Oxide (NO) level

**Fig. 3:**
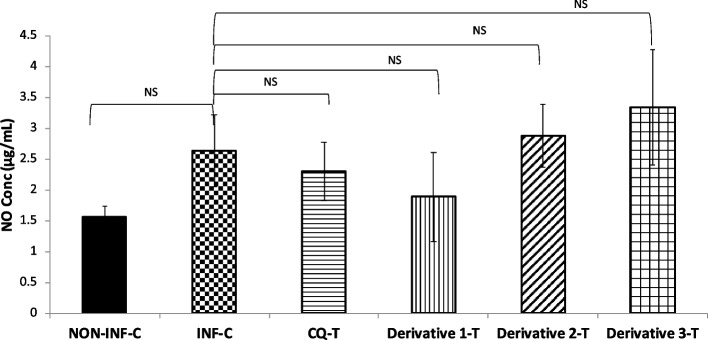
Effect of Chalcone derivatives 1 (10 mg/kg), 2 (20 mg/kg) and 3 (10 mg/kg) using Chloroquine diphosphate (10 mg/kg) as a standard chloroquine sensitive antimalarial drug on nitric oxide level (μg/mL) in *P. berghei* NK-65 infected mouse model. Data are represented as mean ± SEM, *n* = *6* mice. NS: non-significant. Abbreviation Used: NON-INF-C: Non-infected control, INF-C: Infected Control, CQ-T: Chloroquine Treated

Using the Griess reagent, NO production was measured in serum samples from mice sacrificed on the 8^th^ post-infection day. Figure 3 shows that there was no difference between the non-infected and infected control groups and the treated groups.

### Cytokines Level

**Fig. 4:**
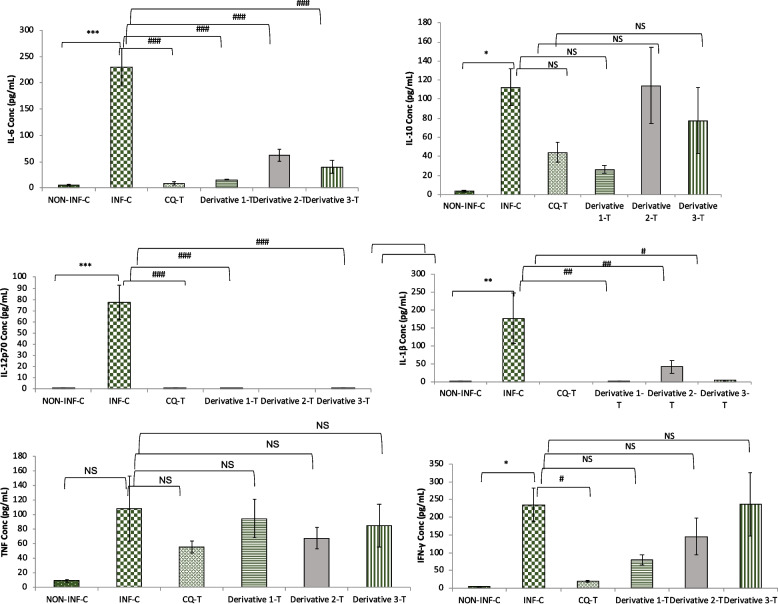
Effect of Chalcone derivatives 1 (10 mg/kg), 2 (20 mg/kg) and 3 (10 mg/kg) using Chloroquine diphosphate (10 mg/kg) as standard chloroquine sensitive antimalarial drug on Cytokines expression (pg/mL) in *P. berghei* NK-65 infected mouse model. Data are represented as mean ± SEM, *n* = *5* mice. *p < 0.05, **p < 0.01, ***p < 0.001 (*Group represents INF-C, CQ-T, Chalcone derivatives 1, 2 and 3 compared to NON-INF-C Control group) and ^#^*p* < 0.05, ^# #^ *p* < 0.01, ^# # #^ *p* < 0.001 (^#^Group represents CQ-T, chalcone derivatives 1, 2, and 3 compared to infected Control group); NS: non-significant. Abbreviation Used: NON-INF-C: Non-infected control, INF-C: Infected Control, CQ-T: Chloroquine Treated

BD CBA Mouse Soluble Protein Flex Set (IL-6, IL-10, IL-12p70, IL-1β, TNF, IFN-γ) analysis revealed significant increase in serum level of cytokines IL-6 (*p* < 0.001), IL-10 (*p* < 0.05), IL-12p70 (*p* < 0.001), IL-1β (*p* < 0.01) and IFN-γ (*p* < 0.01) in infected-control except TNF, which showed non-significant increase as compared to non-infected control. There was a significant (*p* < 0.001) reduction in IL-6 levels in all treated groups as compared to the infected control. The expression of IL-10 and TNF was found to be statistically non-significant as compared to the infected control in all treated groups. The level of IL-12p70 in the CQ-treated (0.76 ± 0.46 pg/mL), derivative 1 (0.36 ± 0.36 pg/mL), and derivative 3 (0.6 ± 0.39 pg/mL) treated groups were significantly lower (*p* < 0.001) when compared to the infected control (77.58 ± 15.31 pg/mL) while no expression was observed in the derivative 2 treated group. The level of IL-1β was significantly decreased in derivative 1 (*p* < 0.01), derivative 2 (*p* < 0.05), and derivative 3 (*p* < 0.01) treated, as compared to the infected control. IFN-γ levels, on the other hand, were only significantly lower in CQ-treated groups when compared to the infected groups (Fig. 4).

### Histopathology

**Fig. 5:**
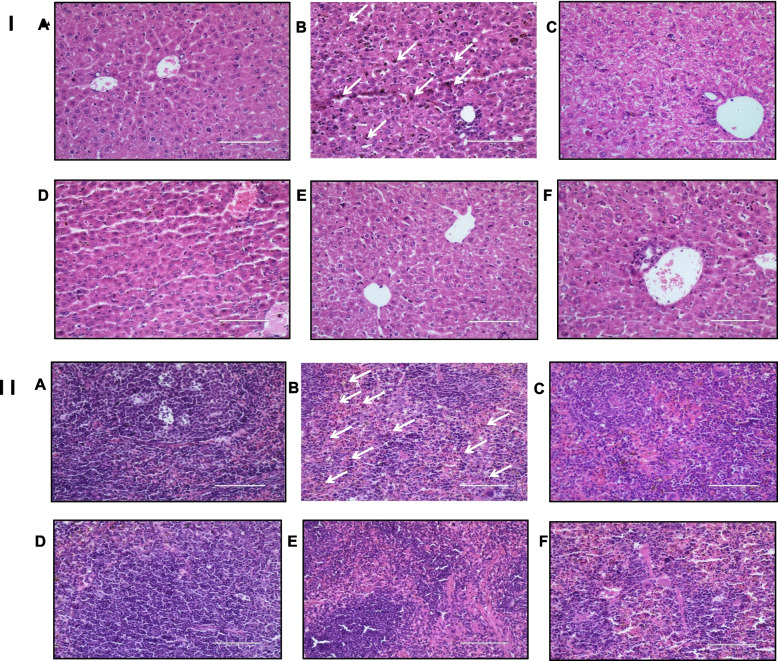
Photomicrographs depicting Hematoxylin & Eosin stained Liver (I) and Spleen (II) sections at 400X magnifications of mice treated with Chalcone derivatives 1 (10 mg/kg), 2 (20 mg/kg) and 3 (10 mg/kg) using Chloroquine diphosphate (10 mg/kg) as standard chloroquine sensitive antimalarial drug in *P. berghei* NK-65 infected mouse model for five days. **A** Non-infected Control, **B** Infected Control **C** Chloroquine-Treated (10 mg/kg) **D** Derivative 1 Treated (10 mg/kg), **E** Derivative 2 Treated (20 mg/kg) and **F** Derivative 3 Treated (10 mg/kg). Arrows in I and II B indicates deposition of hemozoin pigment. Bar scale represent 100 μm

Histopathological sections of the liver showed dilated and congested hepatic sinusoids, dense with hypertrophied Küpffer’s cells, variable mononuclear cells, and pRBC. The hemozoin pigment was widely scattered in Küpffer’s cells in the infected control group. However, the accumulated pigment was comparatively less in all the treated groups as compared to the infected control. Moreover, histologically, the spleen sections showed deposition of hemozoin pigment in the pulp histiocytes and sinusoidal lining cells. Pigment deposition was found to be prominent in the case of infected control as compared to all other treated groups. In CQ-treated mice, derivative 1 and derivative 2-treated groups showed few sites of hemozoin deposition as compared to the derivative 3 treated group (Fig. 5).

### Immunohistochemistry

**Fig. 6:**
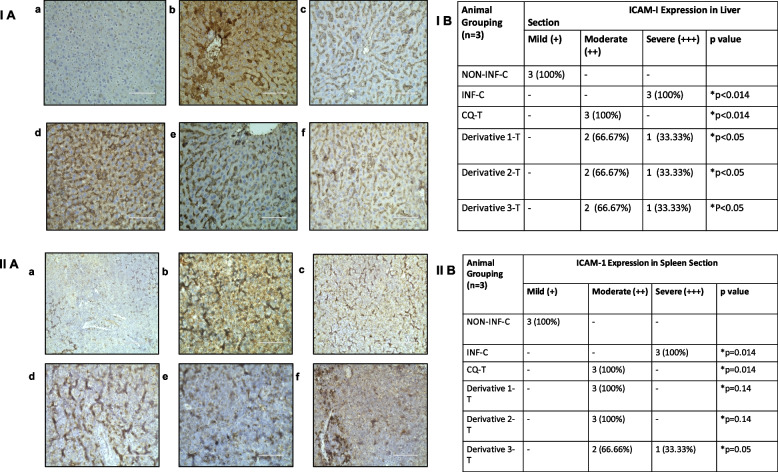
Photomicrographs depicting ICAM-1 stained liver sections at 400X magnifications (I A) and, ICAM-1 stained spleen sections at 200X magnifications (II A) of mice treated with Chalcone derivatives 1 (10 mg/kg), 2 (20 mg/kg) and 3 (10 mg/kg) using Chloroquine diphosphate (10 mg/kg) as standard chloroquine sensitive antimalarial drug in *P. berghei* NK-65 infected mouse model for five days. Biotin-conjugated secondary antibody and streptavidin-conjugated horseradish peroxidase from DAB Substrate kit ( Scy Tek) were applied to sections to amplify the antigen signal for subsequent 3,3-diaminobenzidine staining, which produces a permanent brown color. a) Non-infected Control, b) Infected Control c) Chloroquine-Treated (10 mg/kg) d) Chalcone Derivative 1 Treated (10 mg/kg), e) Derivative 2 Treated (20 mg/kg) and f) Derivative 3 Treated (20 mg/kg). Bar scale represent 100 μm. (I B & II B) ICAM-1 expression in liver section of mice and, ICAM-1 expression in spleen section of mice. Pearson Chi-square, contingency co-efficient, correlations test was applied for statistical significance (*p < 0.05)

Mild ICAM-1 expression in immunostained liver sections was observed in all non-infected control mice (Fig. 6. I A (a)). Percentage ICAM-1 expression is shown (Fig. 6. I B), for each of the six studied groups (*n* = *3*). In mild expression, hepatocytes did not show any ICAM-1 expression and it was only partially expressed in the endothelial lining of sinusoids. Marked ICAM-1 expression was observed in all infected control mice with intense positivity on sinusoidal endothelium as well as hepatocytes (Fig. 6. I A (b)). Moderate expression was observed in sinusoidal endothelium and hepatocytes of CQ treated (Fig. 6. I A (c)), derivative 1, 2, and 3 treated groups (Fig. 6. I (d, e, f)). In the case of immunostained spleen section, mild expression of ICAM-1 was observed in all non-infected control mice (Fig. 6. II A (a)). In contrast, marked expression was observed in all infected control mice (Fig. 6. II A (b)). Moderate expression was shown by the CQ treated, derivative 1, 2, and 3 treated groups (Fig. 6. II A (c, d, e & f)). However, all the mice in CQ treated, derivative 1 and 2 treated showed 100% moderate expression, but there was 66.66% of moderate and 33.33% of marked expression shown in the derivative 3-treated group. The percentage of ICAM-1 expression in immunostained spleen sections is shown (Fig. 6. II B) for each of the six studied groups (*n* = *3*).

### Transmission electron microscopy

**Fig. 7:**
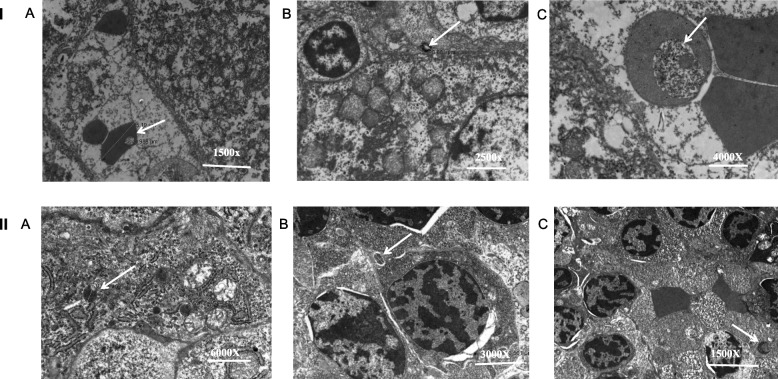
Electron micrograph of (I) Liver section and (II) Spleen section of *P. berghei* infected mouse model depicting effect of Chalcone derivative (10 mg/kg) using Chloroquine diphosphate (10 mg/kg) as standard antimalarial drug in *P. berghei* NK-65 infected mouse model for five days. **A** Infected Control, **B** CQ-Treated (10 mg/kg) and **C**) Chalcone Derivative-Treated (10 mg/kg). Arrows indicates malaria parasite

Numerous pRBC were seen adhered to macrophages and endothelial cells via their surface knobs in the liver and spleen in both the CQ treated group and the chalcone derivative treated (Fig. 7. I (B, C) and II (B, C)).

## Discussion

The in vivo model was recruited for the present study since it demonstrates considerable prodrug effects and also possible engagement of the immune system in the suppression of infection [40]. *P. berghei*-infected mice are thought to be the best model for comparing to human malaria [41]. Furthermore, using rodent models of malaria, a variety of conventional antimalarial agents such as CQ, halofantrine, mefloquine, and, more recently, artemisinin derivatives have been screened and identified [42]. Rane’s test, which assesses the curative potential of a compound on established infections, is frequently employed for antimalarial drug screening [43]. In this protocol, evaluation of the percentage inhibition of parasitemia is the most definite parameter for preliminary screening. In the present study, percentage parasitemia was significantly reduced in all chalcone derivatives treated groups at the dose of 10 mg/kg of derivative 1, 20 mg/kg of derivative 2, and 10 mg/kg of derivative 3, as well as the CQ treated group, which was dosed at 10 mg/kg as compared to the untreated non-infected control group. These results were comparable to licochalcone A, a more active chalcone against *P. falciparum* as compared with previously synthesised ones. Licochalcone A was injected intraperitoneally at 5, 10, and 15 mg/kg twice per day for 3 days, strikingly reducing the parasitemia level in mice infected with *P. yoelii* YM and was also able to reduce the parasitemia level in the treated animals after well-established infection [44]. However, both the present screened chalcone derivatives and Licochalcone were unable to clear the parasites completely but could delay the progression of malaria. Presumably, an extended period of treatment may be required for total clearance of the parasites from the animals having well-defined infections. Apart from this, other chalcone derivatives did not show activity against *P. yoelii* (CQ sensitive) under in vivo conditions [45, 46]. Similarly, 2,4-Dimethoxy-4’-Butoxychalcone, protects mice from lethal infections with *P. berghei* and *P. yoelii*, and controls *P. berghei* infection in rats when administered either orally, intraperitoneally, or subcutaneously once per day for 5 days starting 2 h after infection [27]. The present study is the one of the limited study till date that revealed the curative potential of chalcones in malaria mouse models, as most recent studies evaluated only the prophylactic properties of chalcones, in which chalcones significantly inhibited parasitemia on day four and increased survival times [28, 47, 48].

Body weight loss and body temperature deterioration are the typical attributes of malaria-infected mice [49]. So, ideal antimalarial agents, whether they are obtained from nature, semi-synthetic or synthesised chemically, are anticipated to prevent body weight loss in infected mice due to the rise in parasitemia. Despite the fact that a non-significant decrease in weight and temperature was observed with a decrease in parasitemia level. In contrast to humans, increased parasitaemia levels in rodent models typically result in lower metabolic rates and, as a result, lower body temperatures [50], which might result in death [51]. However, in the present study, there was a non-significant decrease in temperature with a decreasing parasitemia on the 5^th^ day.

Splenomegaly and hepatomegaly are among the common phenomena [52, 53]. Both organs are congested and swollen from the accumulation of the malarial pigment, hemozoin, which leads to discoloration. Also, the existence of numerous deformed red cells triggers the spleen to produce countless phagocytes. Thus, phenomenal hyperplasia is manifested by splenomegaly and, infrequently, hepatomegaly [48]. The present study illustrated the accumulation of the malarial pigment, hemozoin, in all treated and infected control groups. Moreover, treatment with CQ, chalcone derivatives 1, 2, and 3 significantly decreases the weight of the spleen, which suggests the therapeutic potential of chalcones is comparable to existing therapy.

Chalcone derivatives have been extensively known for inhibiting NO synthesis, iNOS and cycloxygenase 2 protein expression in lipopolysaccharide stimulated cells. The structure–activity analysis illustrated that chalcones imposed with substituents that can decrease the electronic density in the B ring, such as chlorine atoms or nitro groups, show much better biological activity and selectivity in the inhibition of nitrite production, and mostly position 2 in ring B seems to be more important [54]. However, chalcones having substituents that increase the electronic density of the B-ring, such as butoxy, methoxy, or dimethylamine groups, had no effect on nitrite production inhibition [55, 56]. In the present study, there was no significant change in nitric oxide generation when non-infected and infected control groups were compared to chalcone-treated groups. This supported the previous findings that group substitution and substituents position on ring B play a role [54].

Further, malaria infections are complex syndromes involving a variety of inflammatory responses that can improve cell-to-cell interaction (cytoadherence), cell stimulation with malaria-derived antigens and toxins, and host-derived factors such as cytokines. Moderate levels of cytokines are good for the host as they cause fever [57]. The present study, revealed the significant up-regulation of pro-inflammatory cytokines, such as, IL-1β, IL-6, IL-10, IFN-γ and non-significant elevation of TNF in infected controls on the 9^th^ day post-infection, showing an association with disease severity that supports previous findings. Furthermore, when compared to the infected-control group, chalcones treated groups (derivative 1, 2, and 3) and CQ showed significant reductions in IL-6 and IL-1 levels, while other cytokines such as IL-1 and IFN- γ levels were comparatively low in CQ treated and derivative 1 treated groups, indicating protection against disease progression and modulating the immune response in the *P. berghei* infected malaria mouse model. Also, a significant reduction in IL-12 levels in all treated groups may be associated with a delay in disease progression, which has been found to be increased in the case of uncomplicated malaria [58, 59]. Elevated levels of TNF were reported during *P. falciparum* infection [60, 61], which was observed in all groups compared to non-infected control, which suggests a protective effect of TNF against parasites [62]. These results propose that expression of these cytokines is not an immediate effect of parasitemia level and also suggest that the differences in cytokine concentrations among different groups may not be due to a direct effect of differences in parasite densities. Nevertheless, the fact that the association between cytokines and parasite densities was different among groups might be a reflection of diverse mechanisms of cytokine regulation depending on various other factors [63].

Inflammatory mediators as well as parasite sequestration may be responsible for the disease severity. It has been suggested that the cytokines up-regulate the expression of adhesion molecules such as ICAM-1 that are involved in the binding of the pRBCs to the vascular endothelium [64]. In the present study, intense ICAM-1 expression was observed in all infected mice, both in hepatocytes, Küpffer’s cells and sinusoidal endothelium in the liver section and in endothelial venules and microphage cells of the spleen, whereas moderate expressions in all CQ and chalcone treated groups were seen, which directly inferred a decrease in disease severity upon treatment. Previously, it was explained that these ICAMs and VCAMs play a major role in the trafficking of leukocytes through normal and inflamed tissue [65]. Moreover, our present study is comparable to previous reported studies which showed cytokine induced activation of endothelial cells such as TNF-a, IL-1β (LPS) known to increase the level of ICAM-1 expression on cultured endothelial cells [66, 67], and here also looking at immune-regulation we have observed the similar facts, as increased expression of TNF, IL-1β and other cytokines in infected control which may augment ICAM-1 expression in the same groups. Persistent immunoreactivity in all treated groups signifies longer treatment duration for complete parasite clearance.

Additionally, pathological changes in these infected murine models before and after treatment were observed microscopically in H&E stained slides to depict a clear picture of disease manifestation. In malaria, the main organs infected are the spleen and liver, which mark the pathophysiology of the disease. Here, we have observed an enlarged, oedematous, and brown, grey, or black colored liver, which may be as a result of the deposition of malaria pigment (hemozoin) in the pulp histiocytes and sinusoidal lining cells of the spleen, and mainly in Küpffer’s cells in the liver section, demonstrating the role of macrophages in parasite clearance. There was also phagocytosis of pRBC and RBC by Küpffer’s cells, endothelial cells, and sinusoidal macrophages. Malarial parasites proliferate inside the RBCs of visceral organs, chiefly in the spleen and liver, and this leads to annihilation of RBCs and loss of cells. The parasite's multiplication also results in the synthesis of pigment, which is left over after the parasite digests the cytoplasm of RBCs. As a result, pigment builds up inside these organs, causing them to darken in color.

Besides, *P. falciparum* is the only species that is sequestered in deep vascular beds as the intraerythrocytic parasite matures to the trophozoite and schizont stages. By doing so, it escapes the clearance mechanisms of the spleen and is thus able to proceed to schizogony. Sequestration results from the cytoadherence of erythrocytes infected with trophozoites and schizonts of *P. falciparum* to vascular endothelium; this cytoadherence requires an interaction between specific parasite ligands and endothelial cell receptors [68]. Therefore in the present study, parasite invasion, sequestration in the vesicular bed, and intense glycogen production in both liver and spleen tissue were observed in electron photomicrography, elucidating escape mechanisms adapted by the parasite to avoid clearance mechanisms in the presence of drug treatment.

## Conclusions

**Fig. 8:**
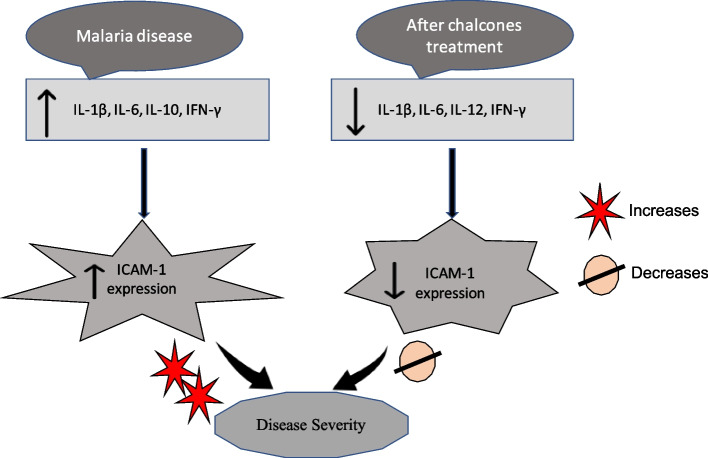
Immunomodulatory Potential of Chalcones

Chalcones have a vast number of bioactive molecules with a wide range of molecular targets. Even slight structural alterations in chalcones can cause them to target different biological functions. Chalcones are a class of chemicals that could potentially be used to generate low-cost, synthetic antimalarial drugs in the future. The present study clearly indicates the potential inhibitory and immunomodulatory action of chalcones against the malarial parasite *P. berghei* (Fig. 8), reporting the effect of chalcones at histological and ultrastructural levels in a malaria mouse model, which mimics the human disease malaria caused by *P. falciparum*. In addition, ICAM-1 expression may play a role in the cytoadhesion of malaria-infected red blood cells or in the recruitment of leukocytes to endothelial activation sites [69]. A decrease in ICAM-1 expression after treatment with chalcone derivatives suggests its protective effect. The study indicates that chalcone derivatives may have some future potential as antimalarial drugs. In the future, these scaffolds and obtained results can be used as a basis to generate more effective leads with potent antimalarial activity. However, further optimization studies are needed in order to improve the bioactivity of these compounds to achieve better curative effects and oral bioavailability. Additionally, the present study lacked a pre-clinical toxicity evaluation that would have provided more clarity on the antimalarial efficacy of the mentioned chalcone derivatives, which is an important part of any drug development process.

## Supplementary Information

**Additional file 1.**

## Data Availability

The datasets used and/or analysed during the current study are available from the corresponding author on reasonable request.

## Abbreviations

*P. falciparum*: *Plasmodium falciparum*
RBC: Red Blood cell
IL-1: Interleukin-1
IL-6: Interleukin-6
IL-8: Interleukin-8
IL-10: Interleukin-10
IL-12 (p70): Interleukin 12(p70)
IFN-γ: Interferon- Gamma
TNF: Tumour Necrosis Factor
NF-kB: Nuclear factor kappa-light-chain-enhancer of activated B cells
VEGF: Vascular Endothelial Growth Factor
ICAM-1: Intercellular Adhesion Molecule 1
VCAM-1: Vascular Cell Adhesion Molecule 1
bcl-2: B-cell lymphoma 2
MMP: Matrix Metalloproteinases
COX: Cyclooxygenase
ROS: Reactive Oxygen Species
CPCSEA: Committee for the Purpose of Control and Supervision of Experiments on Animals
pRBC: Parasitized red blood cells
PBS: Phosphate-buffered saline
CQ: Chloroquine
ABC: Avidin–Biotin–peroxidase complex
DPX: Dibutylphthalate Polystyrene Xylene
OsO_4_: Osmium tetroxide
ANOVA: One-way analysis of variance
SPSS: Statistical Package for the Social Sciences
CQ-T: Chloroquine-Treated
NO: Nitric Oxide
iNOS: Inducible nitric oxide synthase
H&E: Hematoxylin and eosin

## References

[1] Zekar L, Sharman T. Plasmodium Falciparum Malaria. [Updated 2022 Aug 8]. In: StatPearls [Internet]. Treasure Island (FL): StatPearls Publishing; 2022. Available from: https://www.ncbi.nlm.nih.gov/books/NBK555962/. 32310422

[2] Meibalan E, Marti M (2017). Biology of malaria transmission. Cold Spring Harb Perspect Med.

[3] Milner DA (2018). Malaria pathogenesis. Cold Spring Harb Perspect Med.

[4] Popa GL, Popa MI (2021). Recent advances in understanding the inflammatory response in malaria: a review of the dual role of cytokines. J Immunol Res.

[5] World malaria report 2021. Geneva: World Health Organization; 2021. Licence: CC BY-NC-SA 3.0 IGO. Available from: https://www.who.int/teams/global-malaria-programme/reports/world-malaria-report-2021.

[6] Talapko J, Škrlec I, Alebić T, Jukić M, Včev A (2019). Malaria: the past and the present. Microorganisms.

[7] Gelb MH (2007). Drug discovery for malaria: a very challenging and timely endeavor. Curr Opin Chem Biol.

[8] Ashley EA, Phyo AP (2018). Drugs in development for malaria. Drugs.

[9] Flannery EL, Chatterjee AK, Winzeler EA (2013). Antimalarial drug discovery - approaches and progress towards new medicines. Nat Rev Microbiol..

[10] Tajuddeen N, Van Heerden FR (2019). Antiplasmodial natural products: an update. Malar J.

[11] Batovska DI, Todorova IT (2010). Trends in utilization of the pharmacological potential of chalcones. Curr Clin Pharmacol.

[12] Sinha S, Medhi B, Sehgal R (2013). Chalcones as an emerging lead molecule for antimalarial therapy: a review. J Mod Med Chem.

[13] Ouyang Y, Li J, Chen X, Fu X, Sun S, Wu Q (2021). Chalcone derivatives: role in anticancer therapy. Biomolecules.

[14] Cole AL, Hossain S, Cole AM, Phanstiel O (2016). Synthesis and bioevaluation of substituted chalcones, coumaranones and other flavonoids as anti-HIV agents. Bioorg Med Chem.

[15] Mohamad AS, Akhtar MN, Zakaria ZA, Perimal EK, Khalid S, Mohd PA, Khalid MH, Israf DA, Lajis NH, Sulaiman MR (2010). Antinociceptive activity of a synthetic chalcone, flavokawin B on chemical and thermal models of nociception in mice. Eur J Pharmacol.

[16] Yadav VR, Prasad S, Sung B, Aggarwal BB (2011). The role of chalcones in suppression of NF-κB-mediated inflammation and cancer. Int Immunopharmacol.

[17] Jandial DD, Blair CA, Zhang S, Krill LS, Zhang YB, Zi X (2014). Molecular targeted approaches to cancer therapy and prevention using chalcones. Curr Cancer Drug Targets.

[18] Lorusso V, Marech I (2013). Novel plant-derived target drugs: a step forward from licorice?. Expert Opin Ther Targets.

[19] Jantan I, Bukhari SN, Adekoya OA, Sylte I (2014). Studies of synthetic chalcone derivatives as potential inhibitors of secretory phospholipase A2, cyclooxygenases, lipoxygenase and pro-inflammatory cytokines. Drug Des Devel Ther.

[20] Lee JS, Bukhari SN, Fauzi NM (2015). Effects of chalcone derivatives on players of the immune system. Drug Des Devel Ther.

[21] Li R, Kenyon GL, Cohen FE, Chen X, Gong B, Dominguez JN, Davidson E, Kurzban G, Miller RE, Nuzum EO (1995). In vitro antimalarial activity of chalcones and their derivatives. J Med Chem.

[22] Liu M, Wilairat P, Go ML, Liu M (2001). Antimalarial alkoxylated and hydroxylated chalcones [corrected]: structure-activity relationship analysis. J Med Chem.

[23] Go ML, Liu M, Wilairat P, Rosenthal PJ, Saliba KJ, Kirk K (2004). Antiplasmodial chalcones inhibit sorbitol-induced hemolysis of Plasmodium falciparum-infected erythrocytes. Antimicrob Agents Chemother.

[24] Sharma N, Mohanakrishnan D, Shard A, Sharma A, Saima, Sinha AK, Sahal D (2012). Stilbene-chalcone hybrids: design, synthesis, and evaluation as a new class of antimalarial scaffolds that trigger cell death through stage specific apoptosis. J Med Chem..

[25] Smit FJ (2014). Synthesis, in vitro antimalarial activity and cytotoxicity of novel 4-aminoquinolinyl-chalcone amides. Bioorg Med Chem.

[26] Singh A, Rani A, Gut J, Rosenthal PJ, Kumar V (2017). Piperazine-linked 4-aminoquinoline-chalcone/ferrocenyl-chalcone conjugates: Synthesis and antiplasmodial evaluation. Chem Biol Drug Des.

[27] Chen M, Brøgger Christensen S, Zhai L, Rasmussen MH, Theander TG, Frøkjaer S (1997). The novel oxygenated chalcone, 2,4-dimethoxy-4'-butoxychalcone, exhibits potent activity against human malaria parasite Plasmodium falciparum in vitro and rodent parasites Plasmodium berghei and Plasmodium yoelii in vivo. J Infect Dis.

[28] Domínguez JN, de Domínguez NG, Rodrigues J, Acosta ME, Caraballo N, León C (2013). Synthesis and antimalarial activity of urenyl Bis-chalcone in vitro and in vivo. J Enzyme Inhib Med Chem.

[29] Sinha S, Batovska DI, Medhi B, Radotra BD, Bhalla A, Markova N (2019). In vitro anti-malarial efficacy of chalcones: cytotoxicity profile, mechanism of action and their effect on erythrocytes. Malar J.

[30] Sinha S, Radotra BD, Medhi B, Batovska DI, Markova N, Sehgal R (2020). Ultrastructural alterations in Plasmodium falciparum induced by chalcone derivatives. BMC Res Notes.

[31] Chimanuka B, Gabriëls M, Detaevernier MR, Plaizier-Vercammen JA (2002). Preparation of beta-artemether liposomes, their HPLC-UV evaluation and relevance for clearing recrudescent parasitaemia in Plasmodium chabaudi malaria-infected mice. J Pharm Biomed Anal..

[32] Sinha S, Prakash A, Medhi B, Sehgal A, Batovska DI, Sehgal R (2021). Pharmacokinetic evaluation of Chalcone derivatives with antimalarial activity in New Zealand White Rabbits. BMC Res Notes.

[33] Nardos A, Makonnen E (2017). In vivo antiplasmodial activity and toxicological assessment of hydroethanolic crude extract of Ajuga remota. Malar J.

[34] Mekonnen LB (2015). In vivo antimalarial activity of the crude root and fruit extracts of Croton macrostachyus (Euphorbiaceae) against Plasmodium berghei in mice. J Tradit Complement Med.

[35] Sun J, Zhang X, Broderick M, Fein H (2003). Measurement of nitric oxide production in biological systems by using Griess reaction assay. Sensors.

[36] Pascoe S, Gatehouse D (1986). The use of a simple haematoxylin and eosin staining procedure to demonstrate micronuclei within rodent bone marrow. Mutat Res.

[37] Rudin W, Eugstewr HP, Bordmann G, Bonato J, Muller M, Yamage M, Ryffel B (1997). Resistance to cerebral malaria in tumor necrosis factor-alpha-deficient mice is associated with a reduction of intercellular adhesion molecule-1 up-regulation and T helper type 1 response. Am J Pathol.

[38] Wisner-Gebhart AM, Brabec RK, Gray RH (1980). Morphometric studies of chloroquine-induced changes in hepatocytic organelles in the rat. Exp Mol Pathol.

[39] Ryley JF, Peters W (1970). The antimalarial activity of some quinolone esters. Ann Trop Med Parasitol.

[40] Waako PJ, Gumede B, Smith P, Folb PI (2005). The in vitro and in vivo antimalarial activity of Cardiospermum halicacabum L. and Momordica foetida Schumch. Et Thonn. J Ethnopharmacol..

[41] Peters W (1974). Prevention of drug resistance in rodent malaria by the use of drug mixtures. Bull World Health Organ.

[42] Madara A, Jayi JA, Salawu OA, Tijani AY (2010). Anti-malarial activity of ethanolic leaf extract of Piliostigma thonningii Schum (Caesalpiniacea) in mice infected with Plasmodium berghei berghei. African J Biotech..

[43] Box ED, Gingrich WD, Celaya BL (1954). Standardization of a curative test with Plasmodium berghei in white mice. J Infect Dis.

[44] Chen M, Theander TG, Christensen SB, Hviid L, Zhai L, Kharazmi A (1994). Licochalcone A, a new antimalarial agent, inhibits in vitro growth of the human malaria parasite Plasmodium falciparum and protects mice from P. yoelii infection. Antimicrob Agents Chemother..

[45] Tomar V, Bhattacharjee G, Kamaluddin, Rajakumar S, Srivastava K, Puri SK (2010). Synthesis of new chalcone derivatives containing acridinyl moiety with potential antimalarial activity. Eur J Med Chem..

[46] Gutteridge CE, Major JW, Nin DA, Curtis SM, Bhattacharjee AK, Gerena L, Nichols DA (2016). In vitro efficacy of 2, N-bisarylated 2-ethoxyacetamides against Plasmodium falciparum. Bioorg Med Chem Lett.

[47] DomiÃÅnguez JN, LeoÃÅn C, Rodrigues J, de Gamboa DomiÃÅnguez N, Gut J, Rosenthal PJ (2005). Synthesis and antimalarial activity of sulfonamide chalcone derivatives. Farmaco..

[48] Pandey AK, Sharma S, Pandey M, Alam MM, Shaquiquzzaman M, Akhter M (2016). 4, 5-Dihydrooxazole-pyrazoline hybrids: Synthesis and their evaluation as potential antimalarial agents. Eur J Med Chem.

[49] Langhorne J, Quin SJ, Sanni LA (2002). Mouse models of blood-stage malaria infections: immune responses and cytokines involved in protection and pathology. Chem Immunol.

[50] Chinchilla M, Guerrero OM, Abarca G, Barrios M, Castro O (1998). An in vivo model to study the anti-malaric capacity of plant extracts. Rev Biol Trop.

[51] Mengiste B, Mekonnen E, Urga K (2012). In vivo animalarial activity of Dodonaea angustifolia seed extracts against Plasmodium berghei in mice model. MEJS.

[52] Farthing MJG, Rolston DDK, Kumar PJ, Clark ML (1987). Infectious diseases and tropical medicine.. Clinical Medicine.

[53] Bates I, Bedu-Addo G (1997). Chronic malaria and splenic lymphoma: clues to understanding lymphoma evolution. Leukemia.

[54] Wu J, Li J, Cai Y, Pan Y, Ye F, Zhang Y, Zhao Y, Yang S, Li X, Liang G (2011). Evaluation and discovery of novel synthetic chalcone derivatives as anti-inflammatory agents. J Med Chem.

[55] Rojas J, Payá M, Dominguez JN, Luisa FM (2002). The synthesis and effect of fluorinated chalcone derivatives on nitric oxide production. Bioorg Med Chem Lett.

[56] Patil CB, Mahajan SK, Katti SA (2009). Chalcone: a versatile molecule. J Pharm Sci Res.

[57] Depinay N, Franetich JF, Grüner AC, Mauduit M, Chavatte JM, Luty AJ (2011). Inhibitory effect of TNF-α on malaria pre-erythrocytic stage development: influence of host hepatocyte/parasite combinations. PLoS One..

[58] Prakash D, Fesel C, Jain R, Cazenave PA, Mishra GC, Pied S (2006). Clusters of cytokines determine malaria severity in Plasmodium falciparum-infected patients from endemic areas of Central India. J Infect Dis.

[59] Jain V, Armah HB, Tongren JE, Ned RM, Wilson NO, Crawford S (2008). Plasma IP-10, apoptotic and angiogenic factors associated with fatal cerebral malaria in India. Malar J.

[60] Kwiatkowski D, Hill AV, Sambou I, Twumasi P, Castracane J, Manogue KR, Cerami A, Brewster DR, Greenwood BM (1990). TNF concentration in fatal cerebral, non-fatal cerebral, and uncomplicated Plasmodium falciparum malaria. Lancet.

[61] Tchinda VH, Tadem AD, Tako EA, Tene G, Fogako J, Nyonglema P (2007). Severe malaria in Cameroonian children: correlation between plasma levels of three soluble inducible adhesion molecules and TNF-alpha. Acta Trop.

[62] Taverne J, Tavernier J, Fiers W, Playfair JH (1987). Recombinant tumour necrosis factor inhibits malaria parasites in vivo but not in vitro. Clin Exp Immunol.

[63] Ahmed MZ, Bhardwaj N, Sharma S, Pande V, Anvikar AR (2019). Transcriptional modulation of the host immunity mediated by cytokines and transcriptional factors in plasmodium falciparum-infected patients of North-East India. Biomolecules.

[64] Hansen DS. Inflammatory responses associated with the induction of cerebral malaria: lessons from experimental murine models. PLoS Pathog. 2012;8(12):e1003045. 10.1371/journal.ppat.1003045 PMC3531491 23300435

[65] Kukielka GL, Hawkins HK, Michael L, Manning AM, Youker K, Lane C (1993). Regulation of intercellular adhesion molecule-1 (ICAM-1) in ischemic and reperfused canine myocardium. J Clin Invest.

[66] Myers CL, Wertheimer SJ, Schembri-King J, Parks T, Wallace RW (1992). Induction of ICAM-1 by TNF-alpha, IL-1 beta, and LPS in human endothelial cells after downregulation of PKC. Am J Physiol.

[67] Henninger DD, Panés J, Eppihimer M, Russell J, Gerritsen M, Anderson DC (1997). Cytokine-induced VCAM-1 and ICAM-1 expression in different organs of the mouse. J Immunol.

[68] Ho M, Bannister LH, Looareesuwan S, Suntharasamai P (1992). Cytoadherence and ultrastructure of Plasmodium falciparum-infected erythrocytes from a splenectomized patient. Infect Immun.

[69] Cserti-Gazdewich CM, Dzik WH, Erdman L, Ssewanyana I, Dhabangi A, Musoke C, Kain KC (2010). Combined measurement of soluble and cellular ICAM-1 among children with Plasmodium falciparum malaria in Uganda. Malar J.

