# Supplementary figures and images for: Antimalarial and immunomodulatory potential of chalcone derivatives in experimental model of malaria
Source: BMC Complement Med Ther. 2022 Dec 12;22:330. doi: 10.1186/s12906-022-03777-w (PMC9743746; doi:10.1186/s12906-022-03777-w)

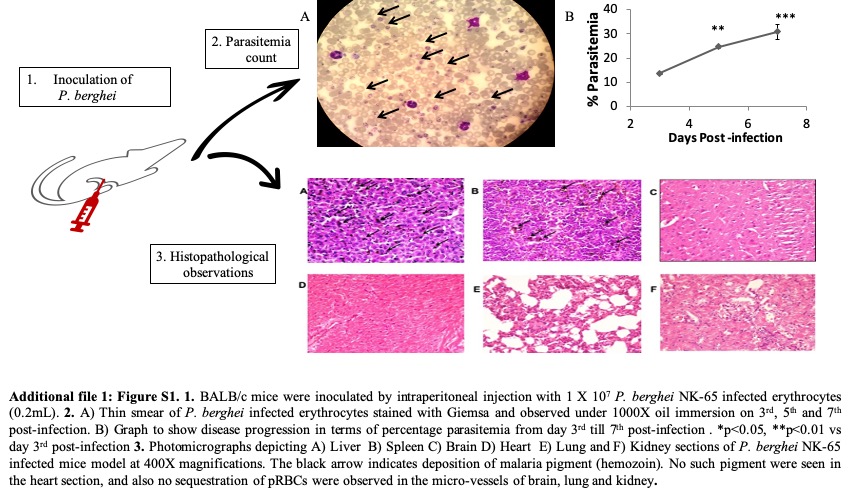

Supplement: Supplementary file 1 — Additional file 1. [file 12906_2022_3777_MOESM1_ESM.jpg]
